# Supplementary material for: Delta-like 1 and Delta-like 4 differently require their extracellular domains for triggering Notch signaling in mice
Source: eLife. 2020 Jan 16;9:e50979. doi: 10.7554/eLife.50979 (PMC6986876; doi:10.7554/eLife.50979)
Supplement: Supplementary file 1. [file elife-50979-supp1.docx]

| **Key Resources Table** | | | | |
| --- | --- | --- | --- | --- |
| **Reagent type (species) or resource** | **Designation** | **Source or reference** | **Identifiers** | **Additional information** |
| Genetic reagent (*Mus musculus*) | *Rosa-CreER* | Seibler et al., 2003 |  |  |
| Genetic reagent (*Mus musculus*) | *iD1* | This paper |  | Conditional Dll1 transgenic mice |
| Genetic reagent (*Mus musculus*) | *iD4* | This paper |  | Conditional Dll4 transgenic mice |
| Cell line (*Homo sapiens*) | HEK293T |  | RRID:CVCL_0063 | Kindly provided by Dr.Yahata, Tokai University School of Medicine  293T cells were used to produce retroviral vectors. |
| Cell line (*Homo sapiens*) | PLAT-E | Abe et al., 2010 | RRID:CVCL_B488 | PLAT-E cells were used to produce retroviral vectors. |
| Cell line (*Mus musculus*) | IDG26.26.10-3 |  |  | Kindly provided by Dr.Kühn, Max Delbrück Center for Molecular Medicine |
| Cell line (*Mus musculus*) | N1/3T3 | Abe et al., 2010 |  |  |
| Cell line (*Mus musculus*) | Lfng-N1/3T3 | Abe et al., 2010 |  |  |
| Cell line (*Mus musculus*) | VF | This paper |  | Mouse BM-derived mesenchymal cell line |
| Cell line (*Mus musculus*) | Dll1-HA/VF | This paper |  | Progenitor: VF  Retrovirally transduced with Dll1-HA |
| Cell line (*Mus musculus*) | D1-D4DSL-HA/VF | This paper |  | Progenitor: VF  Retrovirally transduced with D1-D4DSL-HA |
| Cell line (*Mus musculus*) | D1-D4E1-2-HA/VF | This paper |  | Progenitor: VF  Retrovirally transduced with D1-D4E1-2-HA |
| Cell line (*Mus musculus*) | D1-D4DSL-E1-2-HA/VF | This paper |  | Progenitor: VF  Retrovirally transduced with D1-D4DSL-E1-2-HA |
| Cell line (*Mus musculus*) | D1-D4MNNL-HA/VF | This paper |  | Progenitor: VF  Retrovirally transduced with D1-D4MNNL-HA |
| Cell line (*Mus musculus*) | Dll4-HA/VF | This paper |  | Progenitor: VF  Retrovirally transduced with Dll4-HA |
| Cell line (*Mus musculus*) | D4-D1DSL-HA/VF | This paper |  | Progenitor: VF  Retrovirally transduced with D4-D1DSL-HA |
| Cell line (*Mus musculus*) | D4-D1E1-2-HA/VF | This paper |  | Progenitor: VF  Retrovirally transduced with D4-D1E1-2-HA |
| Cell line (*Mus musculus*) | D4-D1DSL-E1-2-HA/VF | This paper |  | Progenitor: VF  Retrovirally transduced with D4-D1DSL-E1-2-HA |
| Cell line (*Mus musculus*) | D4-D1MNNL-HA/VF | This paper |  | Progenitor: VF  Retrovirally transduced with D4-D1MNNL-HA |
| Cell line (*Mus musculus*) | Dll4-PP-HA/VF | This paper |  | Progenitor: VF  Retrovirally transduced with Dll4-PP-HA |
| Cell line (*Mus musculus*) | OP9 | Yokoyama et al., 2013 | RRID:CVCL_4398 |  |
| Cell line (*Mus musculus*) | Dll1-HA-Tet-Off/OP9 | This paper |  | Progenitor: OP9  Lentivirally transduced with Dll1-HA |
| Cell line (*Mus musculus*) | Dll4-HA-Tet-Off/OP9 | This paper |  | Progenitor: OP9  Lentivirally transduced with Dll4-HA |
| Cell line (*Mus musculus*) | Dll1-HA/OP9 | This paper |  | Progenitor: OP9  Retrovirally transduced with Dll1-HA |
| Cell line (*Mus musculus*) | D1-D4DSL-HA/OP9 | This paper |  | Progenitor: OP9  Retrovirally transduced with D1-D4DSL-HA |
| Cell line (*Mus musculus*) | D1-D4E1-2-HA/OP9 | This paper |  | Progenitor: OP9  Retrovirally transduced with D1-D4E1-2-HA |
| Cell line (*Mus musculus*) | D1-D4DSL-E1-2-HA/OP9 | This paper |  | Progenitor: OP9  Retrovirally transduced with D1-D4DSL-E1-2-HA |
| Cell line (*Mus musculus*) | D1-D4MNNL-HA/OP9 | This paper |  | Progenitor: OP9  Retrovirally transduced with D1-D4MNNL-HA |
| Cell line (*Mus musculus*) | Dll4-HA/OP9 | This paper |  | Progenitor: OP9  Retrovirally transduced with Dll4-HA |
| Cell line (*Mus musculus*) | D4-D1DSL-HA/OP9 | This paper |  | Progenitor: OP9  Retrovirally transduced with D4-D1DSL-HA |
| Cell line (*Mus musculus*) | D4-D1E1-2-HA/OP9 | This paper |  | Progenitor: OP9  Retrovirally transduced with D4-D1E1-2-HA |
| Cell line (*Mus musculus*) | D4-D1DSL-E1-2-HA/OP9 | This paper |  | Progenitor: OP9  Retrovirally transduced with D4-D1DSL-E1-2-HA |
| Cell line (*Mus musculus*) | D4-D1MNNL-HA/OP9 | This paper |  | Progenitor: OP9  Retrovirally transduced with D4-D1MNNL-HA |
| Cell line (*Cricetulus griseus*) | Notch1-hIgG/CHO | Shimizu et al., 1999 |  |  |
| Cell line (*Cricetulus griseus*) | Lfng-Notch1-hIgG/CHO | This paper |  | Progenitor: Notch1-hIgG/CHO  Lentivirally transduced with Lfng |
| Cell line (*Cricetulus griseus*) | Mfng-Notch1-hIgG/CHO | This paper |  | Progenitor: Notch1-hIgG/CHO  Lentivirally transduced with Mfng |
| Cell line (*Cricetulus griseus*) | Notch2-hIgG/CHO | Shimizu et al., 1999 |  |  |
| Cell line (*Cricetulus griseus*) | Lfng-Notch2-hIgG/CHO | This paper |  | Progenitor: Notch2-hIgG/CHO  Lentivirally transduced with Lfng |
| Cell line (*Cricetulus griseus*) | Mfng-Notch2-hIgG/CHO | This paper |  | Progenitor: Notch2-hIgG/CHO  Lentivirally transduced with Mfng |
| Cell line (*Cricetulus griseus*) | Notch1/CHO | Moriyama et al., 2008 |  |  |
| Cell line (*Cricetulus griseus*) | Lfng-Notch1/CHO | This paper |  | Progenitor: Notch1/CHO  Retrovirally transduced with Lfng |
| Cell line (*Cricetulus griseus*) | Mfng-Notch1/CHO | This paper |  | Progenitor: Notch1/CHO  Retrovirally transduced with Mfng |
| Cell line (*Cricetulus griseus*) | Notch2/CHO | Moriyama et al., 2008 |  |  |
| Cell line (*Cricetulus griseus*) | Lfng-Notch2/CHO | This paper |  | Progenitor: Notch2/CHO  Retrovirally transduced with Lfng |
| Cell line (*Cricetulus griseus*) | Mfng-Notch2/CHO | This paper |  | Progenitor: Notch2/CHO  Retrovirally transduced with Mfng |
| Transfected construct (*Mus musculus*) | pCAG-C31Int(NLS)-bpA | Hitz et al., 2007 |  | PhiC31 integrase expression plasmid |
| Transfected construct (*Mus musculus*) | pEx-CAGstop-bpA |  |  | Kindly provided by Dr.Kühn, Max Delbrück Center for Molecular Medicine |
| Transfected construct (*Mus musculus*) | pRMCE-CAG-GFP | This paper |  | Progenitor: pEx-CAGstop-bpA  GFP version of pEx-CAGstop-bpA  (Empty targeting vector for *Rosa26* locus) |
| Transfected construct (*Mus musculus*) | Dll1-HA/pRMCE-CAG-GFP | This paper |  | Progenitor: pRMCE-CAG-GFP  Targeting vector for Cre-dependent Dll1-HA expression |
| Transfected construct (*Mus musculus*) | Dll4-HA/pRMCE-CAG-GFP | This paper |  | Progenitor: pRMCE-CAG-GFP  Targeting vector for Cre-dependent Dll4-HA expression |
| Antibody | FITC-conjugated anti-mouse CD19  (Rat monoclonal) | BioLegend | Cat# 152403  RRID:AB_2629812 | 1:200 |
| Antibody | FITC-conjugated anti-mouse CD44  (Rat monoclonal) | BioLegend | Cat# 103005  RRID:AB_312956 | 1:500 |
| Antibody | FITC-conjugated anti-mouse CD11b  (Rat monoclonal) | BioLegend | Cat# 101205  RRID:AB_312788 | 1:200 |
| Antibody | FITC-conjugated anti-mouse Ly-6G/Ly-6C (Gr-1)  (Rat monoclonal) | BioLegend | Cat# 108405  RRID:AB_313370 | 1:200 |
| Antibody | FITC-conjugated anti-mouse TER119  (Rat monoclonal) | BioLegend | Cat# 116205  RRID:AB_313706 | 1:200 |
| Antibody | PE-conjugated anti-mouse CD19  (Rat monoclonal) | BioLegend | Cat# 152409  RRID:AB_2629838 | 1:200 |
| Antibody | PE-conjugated Hamster IgG Isotype Control  (Armenian Hamster monoclonal) | BioLegend | Cat# 400907  RRID:AB_326593 | 1:200 |
| Antibody | PE-conjugated anti-mouse DLL1  (Armenian Hamster monoclonal) | BioLegend | Cat# 128307  RRID:AB_1133995 | 1:200 |
| Antibody | PE-conjugated anti-mouse DLL4  (Armenian Hamster monoclonal) | BioLegend | Cat# 130807  RRID:AB_1227634 | 1:200 |
| Antibody | APC-conjugated anti-mouse CD11b  (Rat monoclonal) | BioLegend | Cat# 101211  RRID:AB_312794 | 1:1000 |
| Antibody | APC-conjugated anti-mouse Ly-6G/Ly-6C (Gr-1)  (Rat monoclonal) | BioLegend | Cat# 108411  RRID:AB_313376 | 1:1000 |
| Antibody | APC-conjugated anti-mouse c-Kit  (Rat monoclonal) | BioLegend | Cat# 105811  RRID:AB_313220 | 1:200 |
| Antibody | APC-conjugated anti-mouse CD11c  (Armenian Hamster monoclonal) | BioLegend | Cat# 117309  RRID:AB_313778 | 1:200 |
| Antibody | APC-conjugated anti-mouse B220  (Rat monoclonal) | BioLegend | Cat# 103212  RRID:AB_312997 | 1:500 |
| Antibody | APC-conjugated anti-mouse ST-2  (Rat monoclonal) | BioLegend | Cat# 145305  RRID:AB_2561916 | 1:200 |
| Antibody | PE/Cy7-conjugated anti-mouse CD4  (Rat monoclonal) | BioLegend | Cat# 100528  RRID:AB_312729 | 1:500 |
| Antibody | APC/Cy7-conjugated anti-mouse Thy1.2  (Rat monoclonal) | BioLegend | Cat# 105327  RRID:AB_10613280 | 1:1000 |
| Antibody | APC/Cy7-conjugated anti-mouse CD19  (Rat monoclonal) | BioLegend | Cat# 115529  RRID:AB_830706 | 1:200 |
| Antibody | Hamster IgG Isotype Control  (Armenian Hamster monoclonal) | BioLegend | Cat# 400901  Clone: HTK888 | 1:500 |
| Antibody | DyLight 649-conjugated anti-rabbit IgG  (Donkey polyclonal) | BioLegend | Cat# 406406  RRID:AB_1575135 | 1:200 |
| Antibody | PerCP-Cy5.5-conjugated anti-mouse CD25  (Rat monoclonal) | Thermo Fisher Scientific | Cat# 45-0251-82  RRID:AB_914324 | 1:1000 |
| Antibody | APC-conjugated anti-mouse CD8a  (Rat monoclonal) | Thermo Fisher Scientific | Cat# 17-0081-81  RRID:AB_469334 | 1:500 |
| Antibody | APC-conjugated anti-mouse DX5  (Rat monoclonal) | Thermo Fisher Scientific | Cat# 17-5971-81  RRID:AB_469484 | 1:200 |
| Antibody | APC-conjugated anti-mouse PDGFRα  (Rat monoclonal) | Thermo Fisher Scientific | Cat# 17-1401-81  RRID:AB_529482 | 1:200 |
| Antibody | PE/Cy7-conjugated anti-mouse CD45  (Rat monoclonal) | Thermo Fisher Scientific | Cat# 25-0451-81  RRID:AB_2716950 | 1:1000 |
| Antibody | Biotinylated anti-Hamster IgG  (Goat polyclonal) | Thermo Fisher Scientific | Cat# 13-4113-85  RRID:AB_466651 | 1:500 |
| Antibody | PE-conjugated streptavidin | BD Bioscience | Cat# 554061  RRID:AB_10053328 | 1:1000 |
| Antibody | PE-conjugated anti-human IgG  (Goat polyclonal) | Rockland | Cat# 709-1817  RRID:AB_218947 | 1:100 |
| Antibody | Rabbit IgG Isotype Control  (Rabbit monoclonal) | Cell Signaling Technology | Cat# 3900S  RRID:AB_1550038 | 1:2400 |
| Antibody | anti-HA-Tag  (Rabbit monoclonal) | Cell Signaling Technology | Cat# 3724  RRID:AB_1549585 | 1:200 |
| Antibody | HRJ1-5  (Armenian Hamster monoclonal) | Abe et al., 2010 |  | 1:500 |
| Recombinant DNA reagent (plasmid) | pGa981-6 | Abe et al., 2010 |  | TP1-luciferase reporter plasmid |
| Recombinant DNA reagent (plasmid) | pRL-TK | Promega | Cat# E2241 | An internal control plasmid in luciferase reporter assay |
| Recombinant DNA reagent (plasmid) | Dll1-HA/pLevTight-PtTG | Koga et al., 2018 |  | Tet-Off lentiviral vector encoding Dll1-HA |
| Recombinant DNA reagent (plasmid) | Dll4-HA/pLevTight-PtTG | Koga et al., 2018 |  | Tet-Off lentiviral vector encoding Dll4-HA |
| Recombinant DNA reagent (plasmid) | pLevCMV-IP | This paper |  | Lentiviral vector with IRES-Puro |
| Recombinant DNA reagent (plasmid) | Lfng/pLevCMV-IP | This paper |  | Lentiviral vector encoding Lfng |
| Recombinant DNA reagent (plasmid) | Mfng/pLevCMV-IP | This paper |  | Lentiviral vector encoding Mfng |
| Recombinant DNA reagent (plasmid) | pCMV-VSV-G-RSV-Rev | RIKEN BRC | Cat# RDB04393 | Lentiviral packaging plasmid |
| Recombinant DNA reagent (plasmid) | pCAG-HIVgp | RIKEN BRC | Cat# RDB04394 | Lentiviral packaging plasmid |
| Recombinant DNA reagent (plasmid) | MIGR1 | Pui et al., 1999 |  | Retroviral vector with IRES-GFP |
| Recombinant DNA reagent (plasmid) | Dll1-HA/MIGR1 | This paper |  | Progenitor: MIGR1  Retroviral vector encoding Dll1-HA |
| Recombinant DNA reagent (plasmid) | D1-D4DSL-HA/MIGR1 | This paper |  | Progenitor: MIGR1  Retroviral vector encoding D1-D4DSL-HA/MIGR1 |
| Recombinant DNA reagent (plasmid) | D1-D4E1-2-HA/MIGR1 | This paper |  | Progenitor: MIGR1  Retroviral vector encoding D1-D4E1-2-HA |
| Recombinant DNA reagent (plasmid) | D1-D4DSL-E1-2-HA/MIGR1 | This paper |  | Progenitor: MIGR1  Retroviral vector encoding D1-D4DSL-E1-2-HA |
| Recombinant DNA reagent (plasmid) | D1-D4MN-HA/MIGR1 | This paper |  | Progenitor: MIGR1  Retroviral vector encoding D1-D4MN-HA |
| Recombinant DNA reagent (plasmid) | Dll4-HA/MIGR1 | This paper |  | Progenitor: MIGR1  Retroviral vector encoding Dll4-HA |
| Recombinant DNA reagent (plasmid) | D4-D1DSL-HA/MIGR1 | This paper |  | Progenitor: MIGR1  Retroviral vector encoding D4-D1DSL-HA |
| Recombinant DNA reagent (plasmid) | D4-D1E1-2-HA/MIGR1 | This paper |  | Progenitor: MIGR1  Retroviral vector encoding D4-D1E1-2-HA |
| Recombinant DNA reagent (plasmid) | D4-D1DSL-E1-2-HA/MIGR1 | This paper |  | Progenitor: MIGR1  Retroviral vector encoding D4-D1DSL-E1-2-HA |
| Recombinant DNA reagent (plasmid) | D4-D1MN-HA/MIGR1 | This paper |  | Progenitor: MIGR1  Retroviral vector encoding D4-D1MN-HA |
| Recombinant DNA reagent (plasmid) | Dll4-PP-HA/MIGR1 | This paper |  | Progenitor: MIGR1  Retroviral vector encoding Dll4-PP-HA |
| Recombinant DNA reagent (plasmid) | Jag1-HA/MIGR1 | This paper |  | Progenitor: MIGR1  Retroviral vector encoding Jag1-HA |
| Recombinant DNA reagent (plasmid) | Jag2/MIGR1 | Abe et al., 2010 |  | Progenitor: MIGR1  Retroviral vector encoding Jag2 |
| Recombinant DNA reagent (plasmid) | Dll1-hIgG/MIGR1 | This paper |  | Progenitor: MIGR1  Retroviral vector encoding Dll1-hIgG |
| Recombinant DNA reagent (plasmid) | D1-D4DSL-hIgG/MIGR1 | This paper |  | Progenitor: MIGR1  Retroviral vector encoding D1-D4DSL-hIgG |
| Recombinant DNA reagent (plasmid) | D1-D4E1-2-hIgG/MIGR1 | This paper |  | Progenitor: MIGR1  Retroviral vector encoding D1-D4E1-2-hIgG |
| Recombinant DNA reagent (plasmid) | D1-D4DSL-E1-2-hIgG/MIGR1 | This paper |  | Progenitor: MIGR1  Retroviral vector encoding D1-D4DSL-E1-2-hIgG |
| Recombinant DNA reagent (plasmid) | Dll4-hIgG/MIGR1 | This paper |  | Progenitor: MIGR1  Retroviral vector encoding Dll4-hIgG |
| Recombinant DNA reagent (plasmid) | D4-D1DSL-hIgG/MIGR1 | This paper |  | Progenitor: MIGR1  Retroviral vector encoding D4-D1DSL-hIgG |
| Recombinant DNA reagent (plasmid) | D4-D1E1-2-hIgG/MIGR1 | This paper |  | Progenitor: MIGR1  Retroviral vector encoding D4-D1E1-2-hIgG |
| Recombinant DNA reagent (plasmid) | D4-D1DSL-E1-2-hIgG/MIGR1 | This paper |  | Progenitor: MIGR1  Retroviral vector encoding D4-D1DSL-E1-2-hIgG |
| Sequence-based reagent | P1 | This paper |  | 5’-ACGCTTCAAAAGCGCACGTC-3’ |
| Sequence-based reagent | P2 | This paper |  | 5’-AGACGTCGCGGTGAGTTCAG-3’ |
| Sequence-based reagent | P3 | This paper |  | 5’-CGGAGAACCTGCGTGCAATC-3’ |
| Peptide, recombinant protein | Recombinant Murine IL-7 | PeproTech | Cat# 217-17 |  |
| Peptide, recombinant protein | Recombinant Human Flt3-Ligand | PeproTech | Cat# 300-19 |  |
| Commercial assay or kit | Dual-Luciferase® Reporter Assay System | Promega | Cat# E1910 |  |
| Commercial assay or kit | Foxp3 / Transcription Factor Staining Buffer Set | Thermo Fisher Scientific | Cat# 00-5523-00 |  |
| Chemical compound, drug | Tamoxifen | Sigma-Aldrich | Cat# T5648-1G |  |
| Chemical compound, drug | Doxycycline | Clontech | Cat# 631311 |  |
| Software, algorithm | FlowJo | FlowJo, LLC  https://www.flowjo.com/ |  |  |
| Software, algorithm | BD CellQuest Pro | BD Bioscience | RRID:SCR_014489 |  |
| Software, algorithm | AMBER 14 | https://ambermd.org/ |  |  |
